# Using Deep Neural Network to Diagnose Thyroid Nodules on Ultrasound in Patients With Hashimoto’s Thyroiditis

**DOI:** 10.3389/fonc.2021.614172

**Published:** 2021-03-16

**Authors:** Yiqing Hou, Chao Chen, Lu Zhang, Wei Zhou, Qinyang Lu, Xiaohong Jia, Jingwen Zhang, Cen Guo, Yuxiang Qin, Lifeng Zhu, Ming Zuo, Jing Xiao, Lingyun Huang, Weiwei Zhan

**Affiliations:** ^1^ Department of Ultrasound Diagnosis, Ruijin Hospital Affiliated to Shanghai Jiaotong University, Shanghai, China; ^2^ Ping An Technology (Shenzhen) Co., Ltd., Shenzhen, China; ^3^ Computer Centre, Ruijin Hospital Affiliated to Shanghai Jiaotong University, Shanghai, China

**Keywords:** thyroid nodule, ultrasound, deep learning, Hashimoto’s thyroiditis, diagnosis

## Abstract

**Objective:**

The aim of this study is to develop a model using Deep Neural Network (DNN) to diagnose thyroid nodules in patients with Hashimoto’s Thyroiditis.

**Methods:**

In this retrospective study, we included 2,932 patients with thyroid nodules who underwent thyroid ultrasonogram in our hospital from January 2017 to August 2019. 80% of them were included as training set and 20% as test set. Nodules suspected for malignancy underwent FNA or surgery for pathological results. Two DNN models were trained to diagnose thyroid nodules, and we chose the one with better performance. The features of nodules as well as parenchyma around nodules will be learned by the model to achieve better performance under diffused parenchyma. 10-fold cross-validation and an independent test set were used to evaluate the performance of the algorithm. The performance of the model was compared with that of the three groups of radiologists with clinical experience of <5 years, 5–10 years, >10 years respectively.

**Results:**

In total, 9,127 images were collected from 2,932 patients with 7,301 images for the training set and 1,806 for the test set. 56% of the patients enrolled had Hashimoto’s Thyroiditis. The model achieved an AUC of 0.924 for distinguishing malignant and benign nodules in the test set. It showed similar performance under diffused thyroid parenchyma and normal parenchyma with sensitivity of 0.881 *versus* 0.871 (p = 0.938) and specificity of 0.846 *versus* 0.822 (p = 0.178). In patients with HT, the model achieved an AUC of 0.924 to differentiate malignant and benign nodules which was significantly higher than that of the three groups of radiologists (AUC = 0.824, 0.857, 0.863 respectively, p < 0.05).

**Conclusion:**

The model showed high performance in diagnosing thyroid nodules under both normal and diffused parenchyma. In patients with Hashimoto’s Thyroiditis, the model showed a better performance compared to radiologists with various years of experience.

## Introduction

Thyroid cancer has gained much attention because of its rapidly increasing incidence since the last decades though the increase in incidence is partially due to the improvements in diagnosis. It has become the 11th most common cancer in the world and the 5th most common cancer among female worldwide (1–3). Among all thyroid cancer, papillary thyroid cancer (PTC) is the most common histologic type, accounting for 80–90% of all thyroid cancer (4, 5). Hashimoto’s thyroiditis (HT) is the most common auto-immune thyroiditis. The worldwide incidence was reported to range from eight to 46 cases per 1,000 each year depending on different inclusion criteria in various studies. It was at least eight times more prevalent in female, and its incidence is still increasing over time due to social and physical risk factors such as pressure, hormone disorder, and smoking (6, 7). It is considered a risk factor of PTC with an incidence of 0.5–30% in HT patients which is higher than the reported 14.2 per 100,000 person in the general population (4, 8).

Ultrasonography is the most common tool to diagnose thyroid disease, but the accuracy of the diagnosis usually depends on the experience of radiologists. Despite a higher incidence of PTC in patients with HT, it’s more difficult to distinguish between benign and malignant nodules in these patients because they often present a coarse and heterogeneous thyroid parenchyma caused by the repetitive damage of chronic inflammation (9). It was reported that the underlying heterogeneous echogenicity can affect the ultrasound characteristics of thyroid nodule, especially the margin. Microlobulated or irregular margins were more frequently observed among benign nodules under heterogeneous thyroid parenchyma. Since these two features were considered as typical malignant features (10), benign nodules under heterogeneous parenchyma would more likely to be misdiagnosed as malignant nodules, thereby reducing the diagnostic performance of doctors, especially those with less experience. Park et al. (11) reported that in patients with heterogeneous thyroid parenchyma, the accuracy, specificity, and positive predictive rate for diagnosing malignancy were 77.6, 76.3, and 48.7% which were significantly lower than 84.4, 83.7, and 60.9% for patients with homogeneous parenchyma. That means more benign nodules will be misdiagnosed as thyroid cancer. Thus, overdiagnosis and overtreatment are more likely to occur in this part of the population because differential diagnosis between malignant and benign nodules is more challenging in patients with HT.

Computer aided diagnosis (CAD) system has made remarkable progress during these years. From the classic machine learning method (12) to the now prevailing deep learning model, the performance of the CAD system has greatly improved over time. In the traditional machine learning method (13), the explicit features such as size, shape, margin, echogenicity, microcalcification, and macrocalcification were extracted by algorithms or labeled by radiologists, and then sent into the classifiers for training. This kind of expert-knowledge-based system failed to meet the increasing demand for precision, generalization, and efficiency. Recently, deep neural network showed its competency in various tasks for medical image analysis, such as lesion detection and lesion pattern recognition (14, 15). DNN can extract more complex and implicit features and train classifiers synchronously in one unified framework. It can achieve better accuracy and ability of generalization not only because of its huge model capacity but also its deeper and more complex structure. In a recent study with a large training set containing 312,399 images (16), the DNN-based CAD system outperformed most of the radiologists. For these reasons, CAD was considered as a possible solution to reduce overdiagnosis of thyroid cancer. It can overcome the heterogeneity of human radiologists and has shown similar diagnostic performance to human radiologists in many studies (17).

However, no previous studies have been performed to develop a computer aided diagnosis (CAD) system in identifying PTC in HT patients which is believed to be a more challenging task. In this study, we aim to establish a CAD system using deep learning model and test its ability to differentiate malignant and benign thyroid nodules underlying diffused background of HT. Considering the complex heterogeneous echogenicity of thyroid parenchyma in HT patients, we trained and compared two DNN models, one focused only on the interior region of the nodule while another focused not only on the nodule area but also the parenchyma around the nodule. These two models are both pretrained with ImageNet Database.

## Materials and Methods

### Study Design and Inclusion Criteria

This study was a retrospective study approved by the Institutional Review Board, with waiver of informed consent. We retrospectively included 2,932 patients who underwent thyroid ultrasonography from January 2017 to August 2019. 1,666 patients had HT and 1,266 patients had normal thyroid parenchyma. Among all patients, 80% were included as the training set and the rest 20% as the test set so that images in the training set do not appear in the test set.

All selected patients meet the following criteria for image quality control: (1) each nodule should have at least one image from at least two orthogonal planes, (2) the position and size match the ultrasound report and pathological report if pathological result is needed.

The requirement for pathological results depended on the grading of nodules. All nodules were graded using K-TIRADS in this study. Nodules with TIRADS 4A or above need to have definitive pathological results to be included in this study, while nodules graded TIRADS 2 or 3 were recognized as benign nodules and did not necessarily need pathological results.

The inclusion criteria for benign nodules are: (1) nodules graded TIRADS 2 or 3 with or without negative pathological results, (2) nodules graded TIRADS 4A or above with a negative cytological pathology result and Braf mutation verified by repeated FNA, (3) nodules graded TIRADS 4A or above with histological pathology proved to be benign. The inclusion criteria for malignant nodules are malignancy proved by cytological or histological pathology.

The inclusion criteria for HT were as follows: (1) thyroid parenchyma showed heterogeneous echogenicity under ultrasound; (2) serum TPOAb >5.61 IU/ml and/or TGAb >4.11 IU/ml; (3) TRAb within normal range(0–1.75 IU/L).

### Image Acquisition and Evaluation

Ultrasound images were collected by radiologists with at least 3 years’ clinical experience to ensure the quality of images. The ultrasound examinations were performed using MyLab 90, Esaote; iU22, Philips; Resona 7, Mindray; RS 80A Samsung; and Logic E9, GE Healthcare equipped with 7–12 MHz linear-array transducer. The original settings of thyroid mode were used to perform the examination. The region of interest (ROI) of the lesions was annotated using four crossed calipers.

All images included were graded according to K-TIRADS (18). Images in the test set were evaluated by three groups of doctors with clinical experience <5 years, 5–10 years and >10 years respectively. Each group consists of two doctors, and they were asked to give a consensus for whether a nodule was benign or malignant.

### Development of Deep Learning Model

**Figure 1:**
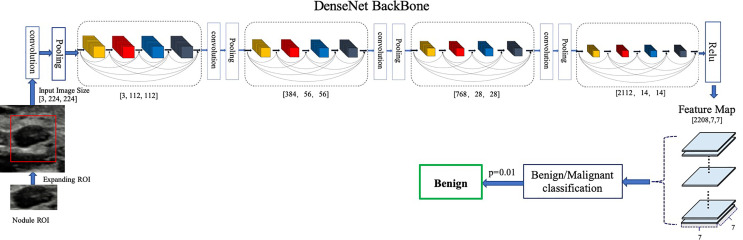
Architecture of our proposed model. DenseNet-161(k = 48) used as the backbone. Different ROI expansions adopted for annotated nodule images of different sizes.

Our proposed model is illustrated in **Figure 1**. We chose DenseNet-161 pretrained with the ImageNet (19, 20) as our model backbone. DenseNet architecture explicitly differentiates between information that is added to the network and information that is preserved. Dense connections with feature maps being concatenated together are used, which are effective for feature exploration, thus DenseNets have made nearly the best performance on the general image classification tasks while substantially reducing the number of model parameters. We used one DenseNet structure with four dense blocks, which extracted features and gradually down-sampled the feature maps, and then input to the full connection layer. Finally, the model outputs the benign probability and the malignant probability of the input image. Then the pathology prediction result, benign or malignant, would be computed according to the probabilities and the threshold value,

We trained a baseline DNN model using only the region of nodule and a modified DNN model using features of both nodule and parenchyma. We expand the annotated nodule ROI according to the rules defined in **Table 1**, letting the model capture more features around the nodule edge and context information. Each ROI was padded with 0 if it reached the image boundary while expanding, and was rescaled without altering the original aspect ratio. To fit the input size of the pre-trained DenseNet-161 model, all training and testing images were resized to 224 × 224.

**Table 1:** Rules of expanding nodule ROI.

Longer side length of nodule ROI	Expanded square ROI size
0 < *len* < 65	*len* +256
65 < *len* < 150	*len* +128
150 < *len* < 256	*len* +64
*len*>= 256	*len* +32

Different ROI expansions adopted for nodule images of different sizes in pixels.

To avoid overfitting, data augmentation is also implemented. We adopted random horizontal flipping, random cropping and rotation within a small range for augmentation because excessive randomization cannot mimic the speckle noise in the ultrasound image. In specific, the range of random translation is not larger than 10% of the longer side length of ROI; the range of random rotation is not bigger than 12.5 angle degrees.

The diffused change information and pathology information on training data were given by the radiologists. Guided by the cross-entropy loss, we can learn the neural network end to end using deep learning framework.

In the training set, we used 10-fold cross validation for the identification of the optimal model, which was then used for the test set classification. We acquired the average classification performance for the test set, plotted in the receiver operating characteristic (ROC) curve. As usually recommended, the optimal threshold value was set at the highest Youden Index, or equivalently, the highest Sensitivity + Specificity (21). Accuracy, sensitivity, specificity, precision, and area under curve (AUC) of ROC curve were extracted from the 10 folds and presented as means ± SD.

Our proposed model was implemented using Python and DL toolkit Pytorch (22). We trained the network with stochastic gradient descent using Adam optimizer with a weight decay rate of 0•0005. All experiments were conducted on two workstations equipped with a 16-core 2.10 GHz Intel Core Processor (Skylake) and two NVIDIA Tesla V100 GPUs.

### Statistical Analysis

General information such as the distribution of sex, age, and percentage of malignancy between training set and test set was calculated and compared between HT and normal groups. The group difference for age was calculated using t test. The group differences for qualitative data such as sex ratio and percentage of malignancy were analyzed using chi-square test.

Accuracy, sensitivity, specificity, precision, and AUC were exploited to evaluate the performance of our model *versus* radiologists. Statistical differences of AUCs between various diagnostic methods were compared using Delong test (23). Mann–Whitney U test was used for the comparison of the model’s specificity, sensitivity, accuracy, and precision between HT subset and normal subset. Chi-square test was used for the comparison between model and radiologists in terms of specificity, sensitivity, accuracy, and precision.

Python was used to perform the Delong test and plot the ROC curve. The rest statistical analysis was performed by SPSS 24.0. p <0.05 was considered statistically significant.

## Results

### Study Population

In total, 2,932 patients with 3,634 nodules and 9,106 images were included in this study. The images were split into training set and test set. All sets were partitioned strictly according to the criteria: images that belonged to the same patient were assigned to the same set. Test set contained 568 patients (710 nodules, 1,805 images) with 332 HT patients (58%). The training set had a total of 2,364 patients (2,924 nodules, 7,301 images), with 1,334 patients (56%) having HT. The baseline characteristics of the training set and test set were listed in **Table 2**.

**Table 2:** Baseline characteristics.

	Training Set	Test Set
**Number of patients, n (%)**	2,364	568
Patients with HT	1,334 (56.4%)	332 (58.5%)
Patients without HT	1,030 (43.6%)	236 (41.5%)
**Number of images, n (%)**	7,301	1,805
Images from patietns with HT	4,128 (56.5%)	1,086 (60.2%)
Images from patietns without HT	3,173 (43.5%)	722 (39.8%)
**Number of nodules, n (%)**	2,924	710
Benign nodules	1,920 (65.7%)	476 (67%)
malignant nodules	1,004 (34.3%)	234 (33%)
**Nodule sizes (cm)**		
Benign nodules	1.09 (0.86)	1.08 (0.89)
malignant nodules	1.08 (0.63)	1.06 (0.61)
**Patient gender, n (%)**		
Male	539 (22.8%)	136 (23.9%)
Female	1825 (77.2%)	432 (76.1%)
**Mean age (years)**	45.29 ± 12.45	45.09 ± 12.41

### Threshold Value and Comparison of Two Deep Neural Network Models

**Figure 2:**
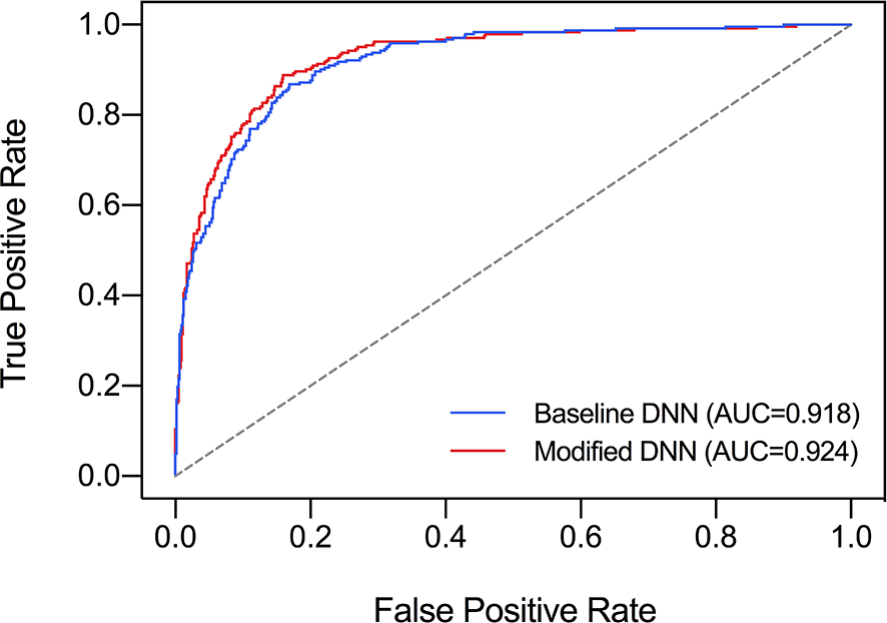
Comparison of ROC curves and AUC of two DNN models. Baseline DNN model learned only the nodule area. Modified DNN model learned the nodule area as well as the surrounding parenchyma.

**Figure 3:**
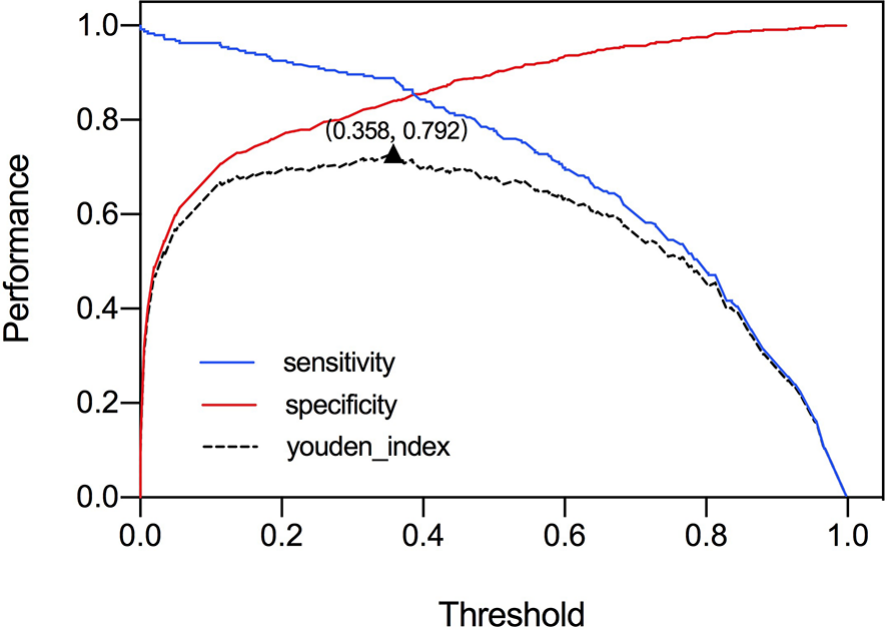
Youden Index and threshold for modified DNN model.

The ROC curve was plotted in **Figure 2** and the corresponding AUC demonstrated the diagnostic performance of our baseline and modified DNN model across all threshold values. The sensitivity, specificity, and Youden Index curve for modified DNN model were mapped in **Figure 3** to show the optimal threshold value. The maximum of Youden Index was 0.729, the corresponding threshold was 0.358. The performance metrics at the optimal threshold were compared between the two models. The AUC, sensitivity, and specificity for the baseline DNN model was 0.918, 0.874, 0.820 compared to 0.924, 0.881, 0.839 for the modified DNN model. The modified model showed a slightly better performance, and therefore we chose the modified DNN model as our CAD model in the following experiments.

### Performance of Deep Neural Network Model on Test Sets

The performance metrics of our DNN model in distinguishing malignant and benign nodules on test set and the two subsets were listed in **Table 3**. It achieved similar AUC under the test set, HT subset and normal subset (AUC = 0.924, 0.924, 0.906 respectively).

**Table 3:** Performance of model in diagnosing malignant nodules on test set and its subsets.

	AUC	Accuracy	Sensitivity	Specificity	Precision
Test set	0.924 (0.006)	0.851 (0.018)	0.881 (0.027)	0.839 (0.031)	0.673 (0.038)
HT subset	0.924 (0.010)	0.852 (0.026)	0.881 (0.035)	0.846 (0.036)	0.540 (0.053)
Normal subset	0.906 (0.010)	0.843 (0.011)	0.871 (0.033)	0.822 (0.029)	0.784 (0.024)
P-Value		0.587	0.938	0.178	<0.01

P-Value is that of diagnostic performance on HT subset versus normal subset; AUC, Areas under the ROC curve. All metrics were the average of 10-fold, presented as Mean (SD).

When comparing the performance between HT subset and normal subset, the model showed similar accuracy, sensitivity, and specificity (p all >0.05). Only precision showed a significant difference (0.540 *vs* 0.784, p < 0.01). When stratified by nodule sizes, listed in **Table 4**, precision showed a notable decrease in the HT subset compared to normal subset among all nodule sizes, and it is more pronounced in nodules <5 mm.

**Table 4:** Performance metrics of DNN model in diagnosing malignant nodules of different sizes, evaluated on normal subset versus HT subset.

		HT Subset	Normal subset
Average size (SD)		0.975 (0.51)	1.25 (0.77)
<5 mm	AUC	0.915	0.895
	Accuracy	0.83	0.825
	Sensitivity	0.859	0.82
	Specificity	0.828	0.826
	Precision	0.327	0.651
5–10 mm	AUC	0.909	0.895
	Accuracy	0.82	0.846
	Sensitivity	0.902	0.868
	Specificity	0.794	0.822
	Precision	0.577	0.841
10–20 mm	AUC	0.883	0.907
	Accuracy	0.832	0.837
	Sensitivity	0.854	0.878
	Specificity	0.824	0.792
	Precision	0.652	0.827
>20 mm	AUC	0.871	0.845
	Accuracy	0.836	0.801
	Sensitivity	0.722	0.724
	Specificity	0.864	0.837
	Precision	0.594	0.688

AUC, Areas under the ROC curve. All metrics were the average of 10-folds.

**Figure 4:**
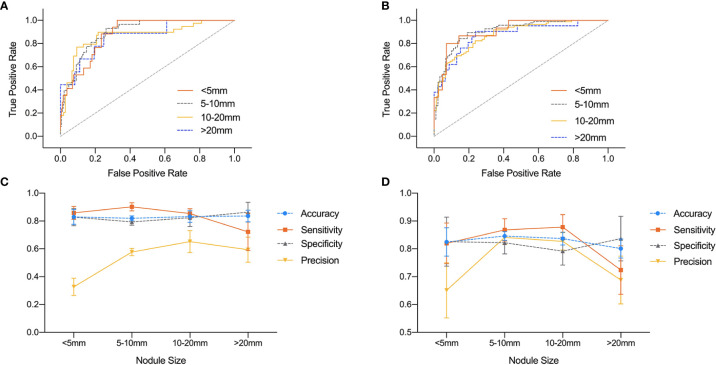
Comparison of ROC curves and performance metrics of DNN model under different nodule sizes. **(A, C)** ROC curves and performance metrics for different nodule sizes under HT subset. **(B, D)** ROC curves and performance metrics for different nodule sizes under normal subset.

The influence of nodule size on DNN model was demonstrated in **Table 4** and **Figure 4**. It was evaluated in normal subset and HT subset respectively. In both subsets, AUC values among nodules <5, 5–10, and 10–20 mm were similar while that for nodules >20 mm was slightly lower. However the ROC curves for nodules with different sizes were quite close as illustrated in **Figure 4**. For both HT subset and normal subset, the accuracy and specificity were similar among different nodule sizes while sensitivity for nodules >20 mm and precision for nodules <5 mm were greatly reduced. What’s more, the precision for nodules >20 mm was also greatly reduced.

### Performance of Deep Neural Network Model Compared to Radiologists Under Diffused Background

**Figure 5:**
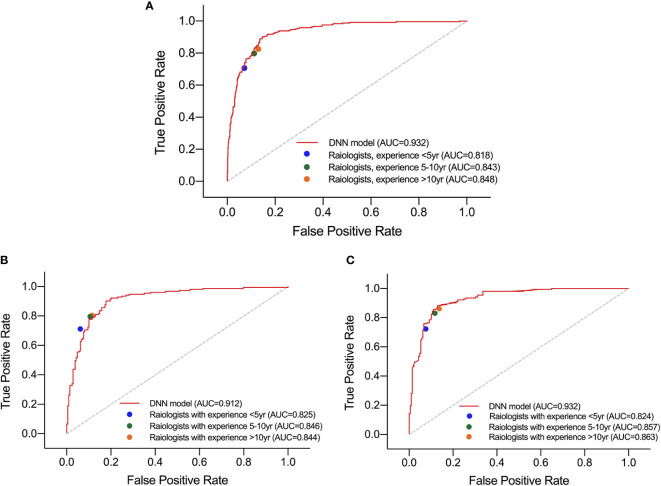
Performance of DNN model and three groups of radiologists in diagnosing malignant nodules under test set **(A)**, normal subset **(B)**, and HT subset **(C)**.

For HT subset, the DNN model achieved a higher AUC than that of the three groups of radiologists which showed significant difference as listed in **Table 5**. However, in the ROC curve (**Figure 5**), the operation points of the radiologists are close to the DNN model’s ROC curve. For the other performance metrics, no significant difference was found for accuracy and precision between DNN model and radiologists. The model showed a higher sensitivity and a lower specificity but significant difference only exist between the DNN model and radiologists with <5 years of experience.

**Table 5:** Performance of model *versus* radiologists of clinical experience <5 years, 5–10 years, and >10 years in diagnosing malignant nodules on the test set and its subsets.

	Diagnostic method	AUC	Accuracy	Sensitivity	Specificity	Precision
Test set	Model	0.924	0.851	0.881	0.839	0.673
	Radiologist <5 yr	0.818	0.868	0.707	0.928	0.784
	Radiologist 5–10 yr	0.843	0.864	0.798	0.888	0.726
	Radiologist >10 yr	0.848	0.858	0.826	0.87	0.701
	P-Value^*^	<0.01	0.781	<0.01	<0.01	0.016
	P-Value^**^	<0.01	1.000	0.001	0.04	0.346
	P-Value^***^	<0.01	0.733	0.777	0.3	0.752
HT subset	Model	0.924	0.852	0.881	0.846	0.540
	Radiologist <5 yr	0.824	0.897	0.723	0.924	0.588
	Radiologist 5–10 yr	0.857	0.875	0.831	0.882	0.514
	Radiologist >10 yr	0.863	0.863	0.862	0.863	0.487
	P-Value^*^	<0.01	0.401	0.001	0.003	0.226
	P-Value^**^	<0.01	0.928	0.060	0.312	0.811
	P-Value^***^	<0.01	0.787	0.486	1.000	0.874
Normal subset	Model	0.906	0.843	0.871	0.822	0.784
	Radiologist <5 yr	0.825	0.842	0.712	0.938	0.893
	Radiologist 5–10 yr	0.846	0.853	0.797	0.894	0.847
	Radiologist >10 yr	0.844	0.85	0.804	0.885	0.837
	P-Value^*^	<0.01	0.603	0	0	0.017
	P-Value^**^	<0.01	0.916	0.01	0.035	0.179
	P-Value^***^	<0.01	0.833	0.015	0.072	0.272

P-Value^*^ is that of model versus radiologist with <5 years’ clinical experience; P-Value^**^ is that of model versus radiologist with 5–10 years’ clinical experience; P-Value^***^ is that of model versus radiologist with >10 years’ clinical experience; AUC, Areas under the ROC curve.

All metrics were the average of 10-folds.

Besides, in the test set and normal subset, the model also showed higher AUC but close operation point on the ROC curve compared to radiologists, as shown in **Table 5** and **Figure 5**. The difference of other metrics between model and radiologists was similar to that under HT subset.

## Discussion

Many studies have achieved satisfied results in nodule diagnosis by using DNN. Buda et al. reported the sensitivity and specificity of a DNN model trained under 1,230 cases (1377 nodules) to be 87 and 52% respectively, which were higher than those of the radiologists with experience ranging from 3 to 32 years and were similar to the consensus of three ACR experts (24). Li et al. included a training set with a total of 42,952 cases which contained the largest sample size so far. The trained DNN model was tested on one internal test set and two external test sets. The AUC of model under three test sets were 0.947, 0.912, and 0.908 respectively, which were significantly higher than those of the six experienced radiologists (16). On the contrary, Gao et al. found that the DNN model performed significantly lower than the radiologists (25). However, they chose AlexNet as their backbone which was different from ours. In our research, DenseNet was chosen as the backbone for its higher performance on the general image classification tasks while substantially reducing the number of model parameters (19, 20).

It is worth mentioning that there was no research revealing the performance of DNN model under diffused thyroid background, and our research filled in this gap. We designed a modified DNN model for diffused background, which learned nodule features as well as background features using border extension. We compared the baseline model which analyzed only the nodule area with our modified DNN model to see how learning thyroid parenchyma helped nodule diagnosis. It turned out that the baseline DNN model showed a slightly lower AUC compared to the modified DNN model. In **Figure 2**, there was an obvious separation between the two ROC curves in the upper left area of the figure which means the modified DNN model had higher sensitivity as well as specificity. This result further supported our hypothesis that learning thyroid parenchyma can help improve the diagnostic accuracy of CNN under heterogeneous background.

The border extension design of the modified DNN model was enlightened by our clinical experience that heterogeneous thyroid parenchyma may affect nodules’ sonographic features. This idea was supported by a series of literatures. Park et al. found that benign nodules in this background are more likely to show vague boundaries (11) which contribute to decrease of accuracy and specificity in differentiating malignant and benign nodules in HT patients. Malignant nodules could also have a more obscure boundary and irregular margin under diffused thyroid parenchyma (26). It could be concluded from the literature that diffused parenchyma affects the nodule’s feature mainly by its border. Therefore, it is reasonable to include parenchyma features around the border using boundary extension so that the influence of parenchyma on nodule border can be considered when diagnosing nodules’ malignancy under diffused background. The rule of ROI expansion we proposed in **Table 1** was based on the fact that a small nodule usually contains less features inside the nodule due to a limited nodule area. Therefore, more border and background information should be taken into consideration during the diagnosis process. For large nodules, there were sufficient features within the nodule area so border information can be less emphasized. What’s more, all images would undergo size normalization process after border expansion before given to the model. For a large nodule, whose image size was already larger than the required input size, the details inside the nodule area would be compressed as image being zoomed out during size normalization. To keep the original sonographic features as unchanged as possible, we should not expand ROI of large nodules too much for it would aggravate the loss of detail.

During the data collection process, our research included HT patients as well as non-HT patients. There are two reasons for this design. First, containing non-HT cases can make our model more generalized. Second, patients with normal parenchyma can serve as a comparison to the HT patients in the test set. Furthermore, the images used in this study were scanned by ultrasound machines from six different companies, which further increased the diversity of data and were closer to the clinical reality.

In patients with HT, the trained DNN model showed a significantly higher AUC value than human but as shown in the ROC curve, dots of radiologists are not too far from the DNN model’s ROC curve. Also, the model showed a higher sensitivity but a lower specificity compared to human radiologists. This indicates that the DNN model outperformed humans in distinguishing malignant and benign nodules mainly due to a higher sensitivity. However in a real-world setting, the overall performance should be considered according to different clinical tasks. Therefore, the model would be more suitable than human radiologists for screening malignancy in a large population especially in HT patients. But in other clinical scenario it may not have that much remarkable advantage compared to human radiologists. Another advantage of DNN model is its diagnostic homogeneity. Ultrasound diagnosis is subjective, and it greatly depends on clinical experience. In patients with HT, the heterogeneous background could affect the margin of nodule and thus further decrease inter-observer and intra-observer agreement, especially between less experienced radiologists (11). On the contrary, DNN model can extract image features quantitatively and output a consistent conclusion through standardized processing methods. Therefore, DNN has a higher reproducibility compared to human radiologists.

However, the precision of our model in the HT subset is lower than in the normal subset which means the trained DNN model is less confident about predicting malignancy within HT subsets. One possible explanation is that the sonograms of benign nodules under HT parenchyma were more suspicious, and thus the model had a higher chance to misdiagnose benign nodules as malignant ones. This hypothesis was supported by a clinical research by Park M. et al. (11) who discovered that benign nodules under HT parenchyma showed more malignant features resulting in a lower positive predictive rate in diagnosing malignancy. Another possible reason is that the nodules in the HT subset are smaller than those in the normal subset. We analyzed the average diameter of nodules under each subset, and we found that the average size of nodules in the HT subset was smaller than that in the normal subset, although not significant. Smaller nodules tend to have less features than big nodules which can cause the model to be less confident in the HT subset. There was also literature supporting the negative effect of nodule size on the performance of model (27) which further supports this hypothesis. It was also notable that the difference of precision between two subsets is more obvious for smaller nodules. This could also be explained by the influence of nodule sizes. Since larger nodules had sufficient features for the model to make reliable predictions, they would be less affected by the heterogeneous parenchyma than the smaller nodules.

The parenchyma had little influence on the performance for our modified DNN model, while the size of the nodules had certain impact on its diagnostic ability. The precision of nodules <5 mm was significantly reduced, while the diagnostic sensitivity of nodules >20 mm was also significantly reduced. As previously reported, Wang et al. also discovered a similar trend (27). One possible reason was that the ROI of small nodules contained less features than big nodules. Therefore, the model was not as confident in the diagnosis of small nodules as in the big ones. The decrease in sensitivity for large nodules might be due to the fact that follicular carcinoma accounts for a greater proportion in nodules >20 mm than in the other three groups. The ultrasound features of follicular carcinoma were similar to benign nodules. However, follicular lesions account for a very low proportion in our training and test sets, and therefore the models didn’t get enough training on identifying this kind of nodule.

Studies have shown that diagnostic accuracy and specificity of doctors in diffused background were reduced (11). In our study, the performance of radiologists didn’t seem to decrease in the HT subset which is contrary to what was reported before. We speculated that one possible reason for this paradoxical situation is that the HT subset may contain slightly more TIRADS 2 and TIRADS 5 nodules due to selection bias when collecting images, which unfortunately made the HT subset relatively easier to diagnose. This was a limitation of our study and could be avoided by stratified sampling according to TIRADS grades in the future study. Another possible reason is that coexisting HT may increase the false negative rate of FNA for subcentimeter thyroid nodules (26). In our study, to avoid false negative cases as much as possible, nodules graded TIRADS 4A or above with a negative cytological results without repeated FNA were eliminated. As a result, there would be a higher chance that subcentimeter nodules graded TIRADS 4A or above in HT patients were excluded. However those nodules are rather difficult to distinguish between malignant and benign. This could also explain why radiologists did better in the HT subset.

This study had several limitations. First, the training set and test set of this study were from the same hospital, lacking external test set. The performance of our model needed to be verified further more by external trials. Second, the data set contained slightly more benign nodules. However, due to the large amount of data used in this study, it should not be considered as a significant deviation. Third, PTC is the main pathological type for malignancy in this study. Only a small portion was follicular carcinoma. The model couldn’t get enough training samples on identifying follicular lesions. Therefore, the model cannot accurately distinguish follicular carcinoma from benign nodules. Another limitation is that the nodules graded as TIRADS 2 and 3 do not necessarily have pathological results. There may be inter-observer variation in nodules with lower TIRADS grading, so it is possible to include very few malignant nodules as benign nodules.

## Conclusion

In conclusion, our modified DNN model performed slightly better than the radiologists with different years of experience in diagnosing thyroid nodules underlying Hashimoto Thyroiditis. It showed higher sensitivity compared to the radiologists. It was also capable of diagnosing malignant nodules in normal patients. Thus, the DNN model might be a possible solution for screening malignant thyroid nodules in the large population.

## Data Availability Statement

The original contributions presented in the study are included in the article/supplementary material. Further inquiries can be directed to the corresponding authors.

## Ethics Statement

This study is a retrospective study and was approved by the Institutional Review Board of Shanghai Ruijin Hospital with waiver of informed consent.

## Author Contributions

YQH, CC, and WZ were major contributors in writing the manuscript, conducting and designing the study. YQH, LZ, WZ, XHJ, JWZ, and WWZ participated in the image annotation, evaluation and study design. QYL, YXQ, CC, LYH, JX, and CG provided technical support. LFZ and MZ provided clinical information on all cases. All authors contributed to the article and approved the submitted version.

## Funding

This research was funded by Smart Health Fund (No. 2018ZHYL0106) by the Shanghai Municipal Health Commission and National Natural Science Foundation of China (Nos. 81671688 and 82071923).

## Conflict of Interest

Authors CC, QYL, CG, YXQ, JX, and LYH were employed by the company Ping An Technology Company of China, Ltd.

The remaining authors declare that the research was conducted in the absence of any commercial or financial relationships that could be construed as a potential conflict of interest.
